# Quantitative detection of *Pf*HRP2 in saliva of malaria patients in the Philippines

**DOI:** 10.1186/1475-2875-11-175

**Published:** 2012-05-25

**Authors:** Andrew O Fung, Robert Damoiseaux, Sarah Grundeen, Jonnas L Panes, Daniel H Horton, Jack W Judy, Theodore B Moore

**Affiliations:** 1 Department of Electrical Engineering, University of California, Los Angeles, USA; 2 Molecular Shared Screening Resources, University of California, Los Angeles, USA; 3 Department of Bioengineering, University of California, Los Angeles, USA; 4, Palawan Baptist Hospital, Roxas, Palawan, Philippines; 5 Biomedical Engineering IDP, University of California, Los Angeles, USA; 6 David Geffen School of Medicine, University of California, Los Angeles, USA; 7 Department of Electrical and Computer Engineering, University of Waterloo, Waterloo, Ontario, Canada

## Abstract

**Background:**

Malaria is a global health priority with a heavy burden of fatality and morbidity. Improvements in field diagnostics are needed to support the agenda for malaria elimination. Saliva has shown significant potential for use in non-invasive diagnostics, but the development of off-the-shelf saliva diagnostic kits requires best practices for sample preparation and quantitative insight on the availability of biomarkers and the dynamics of immunoassay in saliva. This pilot study measured the levels of the *Pf*HRP2 in patient saliva to inform the development of salivary diagnostic tests for malaria.

**Methods:**

Matched samples of blood and saliva were collected between January and May, 2011 from eight patients at Palawan Baptist Hospital in Roxas, Palawan, Philippines. Parasite density was determined from thick-film blood smears. Concentrations of *Pf*HRP2 in saliva of malaria-positive patients were measured using a custom chemiluminescent ELISA in microtitre plates. Sixteen negative-control patients were enrolled at UCLA. A substantive difference between this protocol and previous related studies was that saliva samples were stabilized with protease inhibitors.

**Results:**

Of the eight patients with microscopically confirmed *P. falciparum* malaria, seven tested positive for *Pf*HRP2 in the blood using rapid diagnostic test kits, and all tested positive for *Pf*HRP2 in saliva. All negative-control samples tested negative for salivary *Pf*HRP2. On a binary-decision basis, the ELISA agreed with microscopy with 100 % sensitivity and 100 % specificity. Salivary levels of *Pf*HRP2 ranged from 17 to 1,167 pg/mL in the malaria-positive group.

**Conclusion:**

Saliva is a promising diagnostic fluid for malaria when protein degradation and matrix effects are mitigated. Systematic quantitation of other malaria biomarkers in saliva would identify those with the best clinical relevance and suitability for off-the-shelf diagnostic kits.

## Background

The World Health Organization estimates that in 2009, 225 million people developed malaria, which resulted in 781,000 deaths. Malaria remains endemic in 106 countries despite its heavy burden of fatality and morbidity [1]. Accurate diagnosis of malaria is essential for the avoidance of unnecessary presumptive treatment. Diagnosis by clinical algorithms is most commonly practiced, but varies widely in its accuracy because many malaria symptoms overlap with those of other tropical diseases. Therefore, parasitological diagnosis is recommended in all cases. Examination of Giemsa-stained thick and thin blood smears by light microscopy, a technique introduced in 1904, continues to be the standard for malaria diagnosis because it can quantify parasite density and distinguish between species of *Plasmodium*.

In settings without access to microscopy, antigen-based rapid diagnostic tests (RDTs) can provide a surrogate, albeit qualitative, form of parasite-based diagnosis. First developed in the mid-1990s, RDTs detect parasite antigens from a small volume (usually 5 to 15 *μ*L) of blood using an immunochromatographic assay impregnated on a test strip. The earliest RDTs employed primary antibodies to detect *Plasmodium falciparum* histidine-rich protein 2 (*Pf *HRP2) [2]. Today, commercial tests are tailored to local malaria epidemiology with different combinations of target antigens including genus-specific aldolase, and species-specific HRP2 and parasite lactate dehydrogenase (pLDH).

Notwithstanding the benefits of blood-based tests, their invasive nature requires trained personnel and raises the risk of accidental transmission of infectious diseases. These techniques can also encounter difficulty with patient compliance when frequent blood collection is required from young children and in communities with cultural objections [3].

To overcome the obstacles associated with testing blood, researchers have explored alternative diagnostic media. In contrast to blood, oral fluid presents a reduced biohazard and can be painlessly collected in relatively large quantities by individuals with moderate training. Blood-borne biomarkers that cross from local vasculature into the saliva glands can in principle be detected in oral fluid [4]. Indeed, the diagnostic utility of oral fluid has been demonstrated in immunoassays for infectious diseases such as hepatitis [5], ebola virus [6], measles, rubella [3], and HIV [7].

Biomarkers for malaria have also been identified in saliva. Recent research has correlated levels of anti-malarial IgG between saliva and plasma [8]. Wilson *et al* detected *Pf *HRP2 in whole saliva at 43% sensitivity using a microplate enzyme-linked immunosorbent assay (ELISA) [9]. Using a RDT, Gbotosho *et al* achieved sensitivities of 77.9% in whole saliva and 48.4% in saliva supernatant [10]. In both studies, the accuracy of the assay in saliva was lower than in blood or plasma. These qualitative investigations indicated the potential of saliva-based malaria diagnostics and also highlight the need for more sensitive tests to quantify the range of *Pf *HRP2 in whole saliva.

The development of off-the-shelf saliva diagnostic kits requires further understanding of the availability of biomarkers and dynamics of immunoassay in saliva. These efforts should be supported by best practices for the collection, processing, and storage of samples. The present study reports quantitative detection of *Pf *HRP2 in saliva of malaria patients using a custom chemiluminescent ELISA. A substantive difference between this protocol and previous studies was that saliva samples were stabilized with protease inhibitors. The results of this research will inform design rules for developing rapid diagnostic tests for saliva.

## Methods

### Molecular reagents

Phosphate buffered saline (PBS, P4417), PBS containing 0.05% Tween 20 (PBS-T, 08057), aprotinin (A6279), sodium orthovanadate (Na_3_OV_4_, S6508), and phenylmethanesulfonyl fluoride (PMSF, P7626) were obtained from Sigma-Aldrich. Ninety-six well microtitre plates (Maxisorb 463201, NUNC), blocking buffer (37545, Pierce), and chemiluminescent substrate (SuperSignal ELISA Pico) were obtained from Thermo Fisher Scientific. Dry milk powder (Kroger) was purchased from a local grocer. Bovine serum albumin (RLBSA10) was obtained from Rockland Immunochemicals Inc. Capture antibody (ABMAL-0405) and detector antibody (ABMAL-0401) against *Pf *HRP2 was obtained from Arista Biologicals. Detector antibody was biotinylated (degree of substitution 6.3) using a custom process. Recombinant *Pf *HRP2 was obtained from CTK Biotech (A3000). Peroxidase-labeled streptavidin (SNN2004) was obtained from Invitrogen.

### Site of study and enrollment

**Figure 1:**
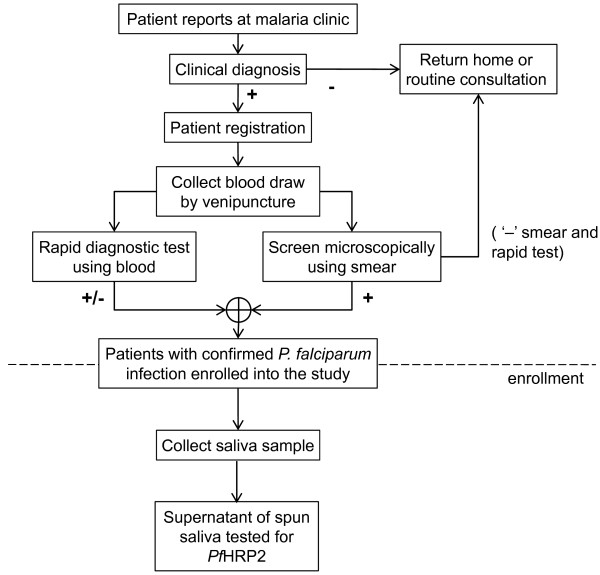
**Enrollment of patients.** Flow chart showing the process for enrollment of patients, collection and analysis of samples.

Matched samples of blood and saliva were collected between January and May, 2011 and processed at Palawan Baptist Hospital (PBH) in Roxas, Palawan, Philippines, after ethical approval by the UCLA Institutional Review Board (IRB Number 09-10-059-02) and the Medical Review Board at PBH. Written consent from the patient, parent, or guardian was obtained before enrollment into the study. The process for enrollment of patients, collection and analysis of samples is shown in Figure 1. Patients were eligible to participate in the study only if *P. falciparum* malaria was microscopically confirmed. Negative-control patients were enrolled at UCLA.

### Diagnosis using thick-film blood smear

Thick-film smears were prepared from blood (venipuncture) at the time of presentation, dried and stained with 10% Giemsa. The smears were inspected for parasites by microscopy under 100× magnification by a pre-qualified expert microscopist. At least 100 parasites and 200 white blood cells were counted. The density of parasites per microliter of blood was calculated with reference to 8,000 white blood cells/*μ*L.

### Rapid diagnostic tests using blood

Rapid diagnostic test kits (OnSite Malaria Pf/Pv Ag, CTK Biotech Inc.) were used according to the manufacturer instructions. A micropipette was used to dispense 10 *μ*L of blood from the sample obtained by venipuncture.

### Collection of saliva samples

Unstimulated oral fluid was collected from each patient. Patients rinsed their mouths with water and expectorated into tubes kept on ice. Up to 5 mL of saliva was collected within 30 min. The saliva was centrifuged at 2600 ×g and 4 °C for 15 min. The supernatant was transferred to a new tube. Aprotinin (0.9 *μ*L, 6.12 Units/mL), Na_3_OV_4_ (3 *μ*L at 400 mM, pH 10 in water), and PMSF (10 *μ*L at 10 mg/mL in isopropyl alcohol) were added per 1 mL supernatant. The samples were stored at -20 °C. Prior to analysis, the samples were thawed, centrifuged and separated again, and 5% v/v 20× PBS-T was added to the supernatant.

### Enzyme-linked immunosorbent assays

**Figure 2:**
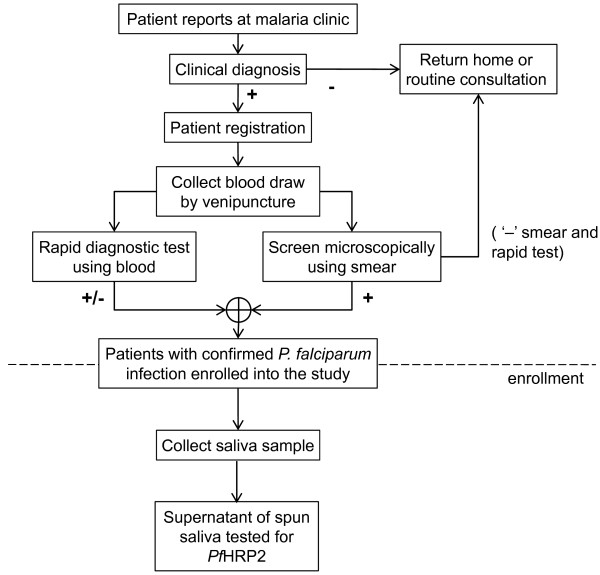
**Configuration of the sandwich ELISA for malaria antigens.** The IgM capture antibody is immobilized on the microtitre plate and binds *Pf *HRP2. The biotinylated IgG detector antibody binds a different epitope of *Pf *HRP2. The use of the biotin-streptavidin system to immobilize peroxidase enzyme provides a degree of amplification. Schematic diagram represents the possibility of multivalent recognition of epitopes by the antibodies.

An antibody sandwich ELISA to detect soluble *Pf *HRP2 (Figure 2) was performed as follows. Microtitre plates were coated by dispensing 50 *μ*L per well of capture antibody at a concentration of 1.25 *μ*g/mL in PBS. The plate was then sealed and incubated for approximately 16 h at 4 °C. The plates were washed three times with PBS-T and blocked using PBS-T containing 4 wt.% dry milk powder for 1 h at room temperature. A blank sample and four calibration solutions of recombinant antigen at concentrations between 0.04 to 5 ng/mL were prepared in either blocking buffer or pooled saliva. The plates were washed three times with PBS-T and incubated with 200 *μ*L per well of sample (spiked buffer for assay development, spiked pooled saliva for calibration samples, or undiluted patient saliva) for 1 h at room temperature with agitation. The wells were then washed five times with PBS-T and incubated with 50 *μ*L per well of biotinylated detection antibody (125 ng/mL in blocking buffer) for 1 h at room temperature under agitation. The plates were washed 5 times with PBS-T and incubated with 50 *μ*L per well of peroxidase-labelled streptavidin (0.8 *μ*g/mL in blocking buffer) for 30 min at room temperature under agitation. The plates were washed five times in PBS-T and reacted with 75 *μ*L per well of chemiluminescent substrate at room temperature for 25 min. The luminescent signal was measured using a microplate luminometer (Victor 3V, Perkin Elmer or Glomax96, Promega Corp.).

The design of the ELISA was optimized across various concentrations of the capture antibody (1.25 to 10 *μ*g/mL), detector antibody (31 to 500 ng/mL), peroxidase-labeled streptavidin (200 to 800 ng/mL), different blocking agents (PBS-BSA, SuperBlock), and incubation conditions (with and without agitation).

### Statistical analysis

Statistical analysis of the ELISA and the patient data was performed in the Stata Analysis and R software environments. Calibration curves for recombinant *Pf *HRP2 were fit with non-linear regression using a four-parameter logistic or power-fit model. Samples yielding ELISA signals below the LOD were reckoned to be zero concentration.

## Results

### Performance of immunoassay and matrix effect of saliva

**Figure 3:**
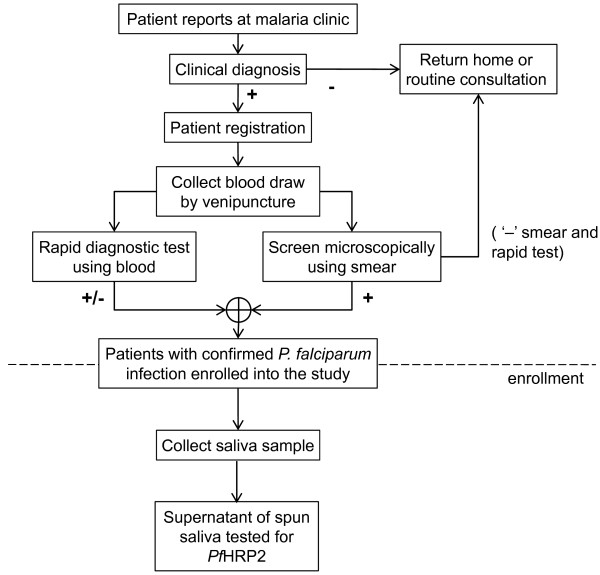
**Typical calibration curves for recombinant*Pf *HRP2 in buffer and saliva.** Relative to buffer, saliva matrix yielded a greater signal at higher concentrations. With the addition of protease inhibitors to saliva, the signal and the LOD were reduced.

A typical calibration curve for recombinant *Pf *HRP2 (Figure 3) in blocking buffer showed a lower statistical limit of detection (LOD; taken at 6 s.d. above the mean of the blank measures [11]) of 173 pg/mL. The assay response was linear (within + /−5%) for concentrations between 163 to 1,506 pg/mL. Relative to buffer, saliva matrix yielded a greater signal at higher concentrations. With the addition of protease inhibitors to saliva, the LOD was 91.7 pg/mL. The detection curve could be adjusted by varying the degree of agitation during incubation and LODs as low as 0.17 pg/mL were achieved. The coefficient of variation (CV) in saliva was less than 25%.

### Collection of blood and saliva samples

Eight thick-film-positive patients and 16 negative-control patients were enrolled. Patient characteristics were collected in addition to the results from the RDT and ELISA (Table 1). Additionally, microscopy and RDT were validated using blood samples from ten healthy volunteers and yielded negative results.

**Table 1:** Data collected from malaria-positive individuals and controls

			**Taking anti-malarial**	**Blood smear *P.f.***	**Axillary temperature**	**RDT for**	**Conc. *Pf*HRP2 by**
**Patient ID**	**Age (yr)**	**Gender (M/F)**	**med. (Y/N)**	**parasite density (/*μ*L)**	**(°C)**	***P.f.***	**ELISA (pg/mL)**
S-01	24	M	N	5,400	37.0	POS	62
S-02	45	M	Y	800	35.7	NEG	1167
S-03	45	F	N	32,000	38.0	POS	538
S-04	23	F	N	1,600	37.2	POS	17
S-05	36	M	N	3,200	35.7	POS	731
S-06	57	M	N	packed field	38.2	POS	479
S-07	15	M	N	6,400	36.1	POS	94
S-08	13	F	N	19,200	35.2	POS	195
S-09	39	M	N	N/A	N/A	N/A	<LOD
S-10	22	F	N	N/A	N/A	N/A	<LOD
S-11	24	M	N	N/A	N/A	N/A	<LOD
S-12	34	M	N	N/A	N/A	N/A	<LOD
S-13	34	M	N	N/A	N/A	N/A	<LOD
S-14	26	M	N	N/A	N/A	N/A	<LOD
S-15	22	F	N	N/A	N/A	N/A	<LOD
S-16	23	F	N	N/A	N/A	N/A	<LOD
S-17	25	M	N	N/A	N/A	N/A	<LOD
S-18	25	M	N	N/A	N/A	N/A	<LOD
S-19	30	F	N	N/A	N/A	N/A	<LOD
S-20	31	F	N	N/A	N/A	N/A	<LOD
S-21	33	F	N	N/A	N/A	N/A	<LOD
S-22	22	M	N	N/A	N/A	N/A	<LOD
S-23	36	F	N	N/A	N/A	N/A	<LOD
S-24	67	M	N	N/A	N/A	N/A	<LOD

Patient characteristics and analysis of blood and saliva specimens. Microscopy of thick-film smears was the gold standard for diagnosis.

### Comparison of microscopy, rapid diagnostic tests, and ELISA

The RDT detected *Pf *HRP2 in the blood of seven of the eight individuals with microscopically confirmed *P. falciparum* malaria. The exceptional sample was drawn from a subject (S-02) already taking anti-malarial medication. Expectedly, this individual had a lower parasite density of 800/*μ*L, which is slightly above the specified detection limit of the OnSite RDT (T-H. Chao, personal communication, 2011). Interestingly, this sample also showed an elevated concentration of salivary *Pf *HRP2. The authors speculate that this result was effected by the medication, perhaps due to rapid and ongoing death of parasites. However, it is unclear why *Pf *HRP2 was not simultaneously elevated in blood and saliva.

Packed fields were observed during microscopy of the blood smear from subject S-06. The parasite density (estimated > 50,000/*μ*L) could not be a accurately determined from the thick-film smear.

**Figure 4:**
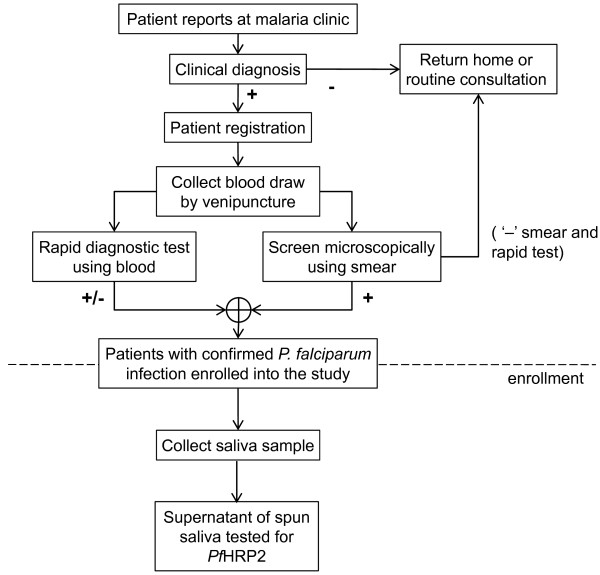
**Estimated concentrations of*Pf *HRP2 in patient saliva.** Example of the estimated concentration of *Pf*HRP2 in patient saliva from one microtitre plate. The standard curve was calculated by fitting calibration samples by non-linear regression. The concentration of *Pf *HRP2 in patient saliva was then estimated from the standard curve.

For each microtitre plate, concentrations of *Pf *HRP2 in the patient saliva were interpolated using standard curves generated from recombinant antigens in pooled saliva (Figure 4). The salivary levels of *Pf *HRP2 ranged from 17 to 1,167 pg/mL.

**Figure 5:**
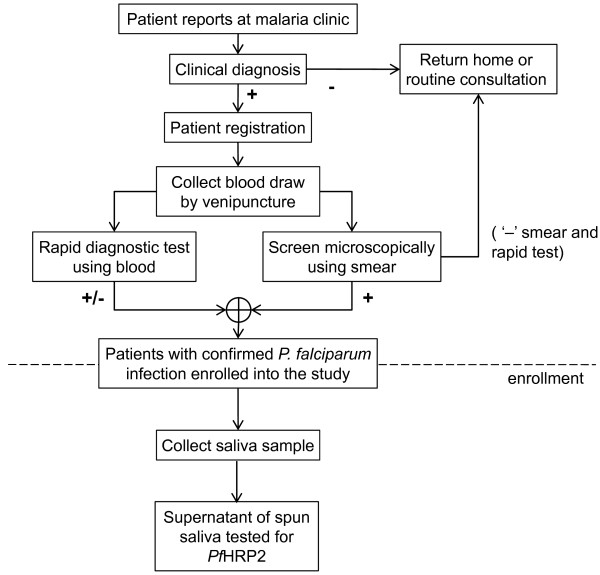
**Salivary levels of*Pf *HRP2.** Aligned dot plot showing the median and interquartile ELISA signals for *Pf *HRP2 levels in malaria-positive patients and negative controls.

The ELISA agreed 100% with the binary outcome of microscopic diagnosis (Table 1). Saliva from negative controls yielded ELISA signals below the LOD. The signals from all saliva samples of malaria-positive patients exceeded the limit of quantification (LOQ; taken at 10 s.d. above the mean of the blank measures) and were markedly higher (Figure 5) than the negative controls (by Welch’s t-test, one-tail *p*=0.021). The difference in populations was further verified by a Wilcoxon rank-sum test (*z*=3.919, *p*<0.001). Using a Monte Carlo simulation of two-tailed ANOVA F tests (2000 iterations, 0.05 significance level), this study achieved a power of 0.842.

## Discussion

There is a growing interest in using saliva as an alternative diagnostic medium to blood because of its relative ease of collection and reduced biohazard. This proof-of-concept study was designed to validate the diagnostic potential of saliva for malaria by quantifying levels of *Pf *HRP2 in clinical samples.

### HRP2 as a biomarker in blood and saliva

*Pf *HRP2 was selected as the target biomarker based on its characterization and precedented use in commercial RDTs. *Pf*HRP2 is a multiplet (*M*_*r*_50 to 85 kDA [12,13]), but antigenically invariant, water-soluble protein that mediates the formation of haemozoin [14]. *Pf*HRP2 is secreted by parasites at all stages, exported through the membrane of the infected red blood cell and then fully released into the blood upon schizont rupture [15].

Since sequestration of parasitized erythrocytes in the host vasculature is a characteristic feature of *P. falciparum* pathology [16], microscopic measurement alone of parasitaemia in peripheral blood could be an inaccurate indicator of the parasite biomass. Methods to measure circulating *Pf *HRP2 were developed to improve estimates of the total parasite burden in cases of extensive sequestration [17]. Qualitative detection of *Pf *HRP2 has become a viable alternative to microscopy to diagnose malaria in remote areas. It is an effective clinical indicator of the severity of past and present parasitaemia. However, the utility of *Pf *HRP2 in monitoring patient response to anti-malarial therapy is limited by the persistence of reactive circulating antigen for several weeks post-treatment [18].

Quantitative studies have measured the levels of *Pf *HRP2 in blood and established its diagnostic significance. Parra *et al* first identified *Pf *HRP2 in the plasma of infected individuals [19]. Other studies reported average levels of *Pf *HRP2 in the range of 1.012 fg per parasite [20] and 8.53 fg per infected RBC [21] in culture medium, and 0.57 to 1.11 *μ*g/mL in plasma [17]. Generally, *Pf *HRP2 is present at higher levels in whole blood than plasma [22,23] and is released in larger quantities than pLDH [21]. The above studies by Desakorn *et al* and Kifude *et al* also reported a correlation between parasite density and plasma levels of parasite antigens.

In the same way, simultaneous measurement of the parasite density and the concentrations of *Pf *HRP2 in plasma and in saliva could reveal a correspondence between the salivary proteome and systemic parasitaemia. Such a study would need to control for the presence of circulating complexed antigen (e.g., with neutralizing IgA) and stage-dependent secretion of *Pf *HRP2 [15]. Furthermore, if *Pf *HRP2 persists in the blood of patients following an active infection, it is also likely that the marker persists in the saliva. These studies are the focus of future work. The correlation between parasite density and salivary *Pf *HRP2 in the present work was not evaluated due to the size of the study cohort (the power of the correlation coefficient was 0.053).

Negative-control samples were not collected from the endemic area due to the possible persistence of *Pf *HRP2 in patients with prior exposure but no active infection. Without complete patient history, one could not discern a patient with persistent antigen from a prior infection from one who had never been infected. The presence of persistent *Pf *HRP2 in the negative controls would have artificially raised the background signal. Since the study measured *Plasmodium* protein and not host response antibody, it was deemed acceptable to recruit negative controls from a non-endemic population.

The results of the ELISA should be interpreted in light of several factors that may complicate true reconciliation of the assay responses to recombinant *Pf *HRP2 and the antigen found in clinical samples. The primary structure of *Pf *HRP2 contains numerous repeated sequences and is, therefore, thought to present multiple epitopes for antibody binding [24]. The degree of multivalence could vary with the size of *Pf *HRP2, which differs among strains. While multivalence enhances the detection signal in immunoassays, the final interpretation is complicated by the genetic diversity of the antigen. Secondly, reports of cross-reactivity [13] suggest that some epitopes on *Pf *HRP2 are also present on the highly homologous *Pf *HRP3. Thus it is possible that the total assay response also included other parasite histidine-rich proteins present in saliva. Finally, as discussed above, the saliva of semi-immune individuals could contain a mixture of free and antibody-bound antigen. Only the free fraction of *Pf *HRP2 would yield ELISA signal.

### Saliva collection

Serum molecules can reach saliva through the gingival crevicular fluid and via mechanisms of intracellular and extracellular transport. The transport of a protein into saliva depends on its molecular mass, solubility, ionization [4], and the salivary pH. Therefore, different molecules can experience varying degrees of dilution during transfer from plasma to saliva. While the precise route followed by *Pf *HRP2 is not known, it most likely enters the saliva duct by pericellular ultrafiltration from the surrounding vasculature. Further investigation into the mechanisms may aid optimization of sample collection.

For analysis by ELISA, robust protocols for the collection and stable storage of saliva samples are important to minimize sample degradation. At room temperature, breakdown of salivary proteins occurs within 30 min of collection [25]. For longer procedures, protein degradation can be mitigated by processing at 4 °C and adding protease inhibitors. Protein degradation may explain why Gbotosho *et al* observed decreased sensitivity for antigen detection in saliva samples that were stored overnight [10].

In the present study, since -80 °C storage was not available in the field, all samples were stored at -20 °C and used within 14 days. The single freeze-thaw cycle was used to denature mucins and improved their separation by centrifugation [26]. The addition of Tween 20 surfactant to the saliva reduced non-specific binding in the immunoassay.

Complex sample preparation and handling are not amenable to a low-cost rapid test. However, it is expected that short (i.e., under 30 min) analyses of fresh samples would largely circumvent problems of degradation. The removal of mucins could be accomplished by extracting the saliva from a sponge collector [27]. The integration of such sample preparation would further enable simple processing for saliva rapid tests.

### Enzyme-linked immunosorbent assay

Whereas diagnostic development requires absolute quantitation of salivary antigens, previous field studies have only reported qualitative detection using commercial tests designed for higher levels of antigen in blood or plasma [9,10,17,20]. Rapid diagnostic tests that rely on the accumulation of gold particles in lateral-flow strips do not achieve a sufficiently low limit of detection for use with saliva samples. Wilson *et al* drew similar conclusions about colorimetric microplate assay kits, i.e., Malaria Ag CELISA, which has reported LODs of 1.5 to 3.91 ng/ml [15,20]. By comparison, an assay suitable for saliva requires a greater signal-to-noise ratio, a lower detection range, and mitigation of matrix effects.

To meet these requirements, this study developed a more sensitive custom chemiluminescent [28] ELISA for *Pf *HRP2 (Figure 2). The pair of antibodies was pre-validated by the vendor for sandwich ELISA. Amplification of the signal was achieved using biotinylated detector antibody with the strong tetravalent binding of the streptavidin-enzyme conjugate. The resulting readout yielded a high signal-to-noise ratio (i.e., compared to colorimetric assays) so that the effect size, *d*, between positive and control populations was very large (*d*=7). This allowed significant conclusions to be drawn despite the limited number of subjects.

It was important to match the detection range of the ELISA with respect to clinical analyte levels. In the absence of prior reports about the physiological levels of *Pf *HRP2 in saliva, it was inferred that salivary concentrations of *Pf *HRP2 would be in the range of 10^1^ to 10^2^ pg/mL based on typical plasma:saliva ratios of protein. Agitation of the samples during incubation provided a useful degree of freedom in the field to tune the detection for clinical samples. The predicted levels of salivary *Pf *HRP2 were indeed supported by the results of the ELISA. Since the enrollment process of this study favoured symptomatic subjects with relatively high parasite densities, further investigation is required to determine the lowest parasite density that yields detectable salivary *Pf *HRP2.

The numerous components of saliva matrix aside from the analyte can have a considerable impact on the performance of the ELISA. Collective matrix effects of binding proteins, drugs, degrading enzymes, and heterophilic antibodies, etc. can differ between binding systems [29]. A common approach to mitigate the matrix effects of saliva is to dilute the sample in a more tractable buffer [30] and measure it against calibration samples prepared in the same buffer. The dilution of saliva samples with PBS was evaluated, but the minimum required dilution was deemed unsuitable for the detection limits that were required for this investigation. Due to the variation of recovery rates in saliva relative to buffer, it was decided to prepare calibration standards in pooled saliva from malaria-negative donors. Calibration standards were included on each microtitre plate to account for inter-plate variation.

The protease inhibitors added to saliva block the activity of serine proteases, tyrosine phosphatases, and alkaline phosphatases. Their collective reactivity significantly reduced the signal and background of the detection curve (Figure 3). Clearly, the assay performance needs to be carefully assessed in the presence of inhibitors or any other additives involved in the collection procedure.

### Design guidelines for saliva immunodiagnostics

Saliva has garnered significant attention as a diagnostic medium for systemic disease, and is particularly attractive as a low-cost, non-invasive approach to meet health needs in developing countries [3,31]. As the number of investigations towards such applications is expected to increase, guidelines are offered for the early-stage development of saliva diagnostics.

When selecting a biomarker of systemic disease, one should begin with a short list of those whose detection is well-characterized in blood or its derivative components. Proteins transferred from blood to saliva may be diluted by up to 100,000×, but the dilution factor is not constant for all analytes [32]. Thus, the physiological range of the target biomarker in saliva should be determined. For pilot studies, physiological levels may not be known *a priori*, and available assay kits for serum measurements could lack the sensitivity to detect the biomarker in saliva. Such cases may require the design of a new assay with a suitable detection range.

The matrix effect of saliva on the assay response should be characterized. If the assay is sufficiently sensitive, then the saliva could be diluted. An alternative strategy is to reduce the sample viscosity by removing mucous with mechanical filtration, or chemical digestion by an *in-vitro* mucolytic agent (e.g., N-Acetyl Cysteine) [33]. Non-specific binding can be mitigated by the addition of detergent or a competitive binding molecule. When undiluted saliva is assayed, it would also be useful to prepare calibration standards in a matrix that yields a consistent recovery rate.

The authors further recommend that the collection of oral fluid should be detailed because this can significantly affect the composition of the sample. For example, gingival cervicular fluid differs markedly from saliva, which can differ yet depending on whether a specific gland was targeted and whether the collection was stimulated or resting. Where possible, fresh saliva should be used and kept on ice after centrifugation. If analysis is to be done at a later date, the samples should be refrigerated and stabilized with appropriate inhibitors.

## Conclusions

The present work detected and quantified *Pf *HRP2 in the saliva of individuals with *P. falciparum* malaria. These findings provide impetus for further investigation of the presence of the *Plasmodium* proteome [34] in host saliva. Future work will measure the concentration gradient of biomarkers between blood and saliva with correlation to parasite density. It will be useful to compare the lower limit of detection in saliva with those achieved by microscopy, lateral flow RDTs, and polymerase chain reaction (PCR). Systematic quantitation of other malaria biomarkers in saliva would identify those with the greatest clinical relevance and diagnostic accessibility. For example, human LDH and aldolase are present in the saliva [25], which suggests that the corresponding *Plasmodium* proteins could be detected there as well.

Saliva is a promising diagnostic fluid for malaria when protein degradation and matrix effects are mitigated. If the burdens of training and instrumentation can be alleviated with automated, portable and sensitive assays, the use of saliva can enable a cost-effective approach for the screening of large populations to enable eradication programs to shift from passive to active surveillance and case management.

## Competing interests

The authors declare that they have no competing interests.

## Author’s contributions

AF, RD, TM, DH conceived and designed the experiments. AF, JP, performed the experiments. AF, SG, analysed the data. RD, DH, JJ contributed reagents/materials/analysis tools. AF wrote the manuscript, which was read and edited by all authors.
